# Characteristics of PCDD/Fs in PM_2.5_ from emission stacks and the nearby ambient air in Taiwan

**DOI:** 10.1038/s41598-021-87468-5

**Published:** 2021-04-14

**Authors:** Shih Yu Pan, Yi Ting Liou, Moo Been Chang, Charles C.-K. Chou, Tuan Hung Ngo, Kai Hsien Chi

**Affiliations:** 1 Institute of Environmental and Occupational Health Sciences, National Yang Ming Chiao Tung University, Taipei, 112 Taiwan; 2 School of Medicine, National Yang Ming Chiao Tung University, Hsinchu, 300 Taiwan; 3 Graduate Institute of Environmental Engineering, National Central University, Chungli, 320 Taiwan; 4 Research Center for Environmental Changes, Academia Sinica, Taipei, 115 Taiwan; 5 International Health Program, National Yang Ming Chiao Tung University, Taipei, 112 Taiwan

**Keywords:** Environmental sciences, Chemistry, Engineering

## Abstract

This study aimed to find the characteristics of polychlorinated dibenzo-p-dioxins and dibenzofurans (PCDD/Fs) in fine particulate matter from different stationary emission sources (coal-fired boiler, CFB; municipal waste incinerator, MWI; electric arc furnace, EAF) in Taiwan and the relationship between PM_2.5_ and PM_2.5_-bound PCDD/Fs with Taiwanese mortality risk. PM_2.5_ was quantified using gravimetry and corresponding chemical analyses were done for PM_2.5_-bound chemicals. Mortality risks of PM_2.5_ exposure and PCDD/Fs exposure were calculated using Poisson regression. The highest concentration of PM_2.5_ (0.53 ± 0.39 mg/Nm^3^) and PCDD/Fs (0.206 ± 0.107 ng I-TEQ/Nm^3^) was found in CFB and EAF, respectively. Higher proportions of PCDDs over PCDFs were observed in the flue gases of CFB and MWI whereas it was reversed in EAF. For ambient air, PCDD/F congeners around the stationary sources were dominated by PCDFs in vapor phase. Positive matrix factorization (PMF) analysis found that the sources of atmosphere PCDD/Fs were 14.6% from EAF (r = 0.81), 52.6% from CFB (r = 0.74), 18.0% from traffic (r = 0.85) and 14.8% from MWI (r = 0.76). For the dioxin congener distribution, PCDDs were dominant in flue gases of CFB and MWI, PCDFs were dominant in EAF. It may be attributed to the different formation mechanisms among wastes incineration, steel-making, and coal-burning processes.

## Introduction

PM_2.5_ exposure could lead to adverse health impacts^1–4^. Studies found association between monthly PM_2.5_ levels and all-cause mortality, death caused by cardiovascular (CVD) and respiratory diseases^5,6^. Significant correlation between PM_2.5_ and hospitalization of asthma, arrhythmia, and myocardial infarction was also found in previous research^7^. Quantitatively, each 10 μg/m^3^ increase of PM_2.5_ concentrations lead to increment of all-cause mortality (1.18%), CVD (1.03%-1.76%), and respiratory disease deaths (1.71%)^8^. Retainment of fine particles in the lungs could cause inflammations^9^ which are enhanced by some PM_2.5_-bound chemicals.

Polychlorinated dibenzo-p-dioxin and furans (PCDD/Fs) were persistent organic pollutants (POPs) announced by United Nations Environment Programme (UNEP). PCDD/Fs have long half-life and flexible mobility in the atmosphere. Atmospheric PCDD/Fs by dry and wet deposition could land on the topsoil surface and eventually through the food chain entered the human body. Oh, et al.^10^ found PCDD/Fs of municipal waste incinerator at atmospheric and soil area in Korea to be 0.66 pg I-TEQ/m^3^ (35.6 pg/m^3^) and 19.1 pg I-TEQ/g (1077.11 pg/g). Yu et al.^11^ found PCDDs in the electric arc furnace (EAF) plant to be dominated by 2,3,4,6,7,8-HpCDD and OCDD when PCDFs were dominated by 1,2,3,4,6,7, 8-HpCDF and OCDF. The result of atmospheric PCDD/Fs concentration was ranged from 0.088 and 13.9 pg I-TEQ/m^3^ nearby the waste incinerators in China^12^. In Taiwan, the annual PM_2.5_ average concentration were found 21.4, 20.2 and 19.9 μg/m^3^, followed a decreased trend^13^. As mentioned the seasonal gas-particle partitions of PCDD/Fs on ambient air in 2017. The gas-particle partitions of PCDD/Fs were 82.2 ~ 90.8% contributed by gas phase on summer^13^. Previous studies observed PCDD/Fs emission from EAF was higher than municipal waste incinerators^14,15^. To the best of our knowledge, limited studies were done to simultaneously evaluate the relationship between stack and ambient PCDD/Fs and PM_2.5_. Most of researches done so far focused on municipal waste incinerators^16–18^ and found the complexity of contributors (mobile sources or other stationary sources) to the ambient air pollution in the vicinity. Therefore, we suggested the use of receptor model to find the possible emission sources and their contributions. According to the inventory of PCDD/Fs showed that incinerators (19.4%) and steelmaking process (54.6%) was the major source of emission. The major PCDD/F emission in Taiwan were from stationary sources including boiler combustion (24.1%), fugitive emission sources (20.8%), sinter plant (15.3%) and electric arc furnaces (14.4%)^19^. Because of the ambient air particles were emitted from different stationary sources. To protect the environment that rule the guideline for air pollutants of PCDD/Fs in emission sources (dioxin emission standards for stationary pollution sources were 1.0 ng I-TEQ/m^3^ for old sources and 0.5 ng I-TEQ/m^3^ for new sources by Environmental Protection Administration in Taiwan, respectively). Therefore, it is crucial to understand the characteristics of this source of emission. In this study, we monitor PCDD/Fs and PM_2.5_ emitted from stationary sources and atmospheric measurements in the vicinities. We also aim to study the relationship between PM_2.5_ and PM_2.5_-bound PCDD/Fs with Taiwanese mortality risk.

## Results

### Mass concentrations of PM_2.5_, PCDD/F levels and chemical compounds in the flue gas of different stationary sources

Highest concentration of PM_2.5_ was found in CFB flue gas at 0.53 ± 0.39 mg/Nm^3^ (n = 5). The flue gas average concentration of PM_2.5_ were 0.34 ± 0.06 and 0.35 ± 0.12 mg/Nm^3^ in MWI (n = 3) and EAF (n = 3), respectively. In flue gas of CFB, the average PCDD/Fs concentrations were 0.003 ± 0.003 and 0.0005 ± 0.0003 ng I-TEQ/Nm^3^ in vapor and solid phase, respectively (Table 1). In MWI flue gas, the average PCDD/F concentrations were 0.021 ± 0.011 and 0.004 ± 0.002 ng I-TEQ/Nm^3^ in vapor and solid phase, respectively. The highest concentrations of PCDD/F were found in EAF flue gas, the average concentrations were 0.204 ± 0.071 and 0.001 ± 0.0003 ng I-TEQ/Nm^3^ in vapor and solid phase, respectively. All of flue gas samples were lower than the emission standards for stationary sources in Taiwan (CFB: 1.0, MWI: 0.1, EAF: 0.5 ng I-TEQ/Nm^3^). The lowest PCDD/F concentrations measured in CFB flue gas maybe attributed to the sulfur content in coal the fuel of CFB. However, previous study found PCDD/Fs from coal combustion to be relatively low^20^. Research of Tuppurainen et al.^21^ found how phenolic precursors converted into sulfuric compounds (ex: dibenzothianthrene and dibenzthiophene) which were similar to PCDD/Fs. Ogawa et al.^22^ and Tuppurainen et al.^21^ elucidated the mechanism of inhibiting PCDD/F formation by adding sulfur.

**Table 1:** Concentration of PM_2.5_, PCDD/Fs and chemical compounds measured in flue gas at different facilities.

Emission sources	CFB (n = 6)	MWI (n = 6)	EAF (n = 6)
TPM (mg/Nm^3^)	3.73	1.24 ± 0.49	1.64 ± 0.32
PM_2.5_ (mg/Nm^3^)	0.53 ± 0.39	0.34 ± 0.06	0.35 ± 0.12
PCDD/Fs (pg I-TEQ/Nm^3^)			
TPM	2.0	5.0 ± 1.0	4.0 ± 2.0
PM_2.5_	0.5 ± 0.3	4.0 ± 2.0	1.0 ± 3.0
Vapor phase	3.0 ± 3.0	21 ± 11	204 ± 71
OC (μg/m^3^)	50.3 ± 37.8	3.57 ± 1.39	7.94 ± 2.20
EC (μg/m^3^)	64.6 ± 30.1	0.912 ± 0.994	1.59 ± 1.84

**Figure 1:**
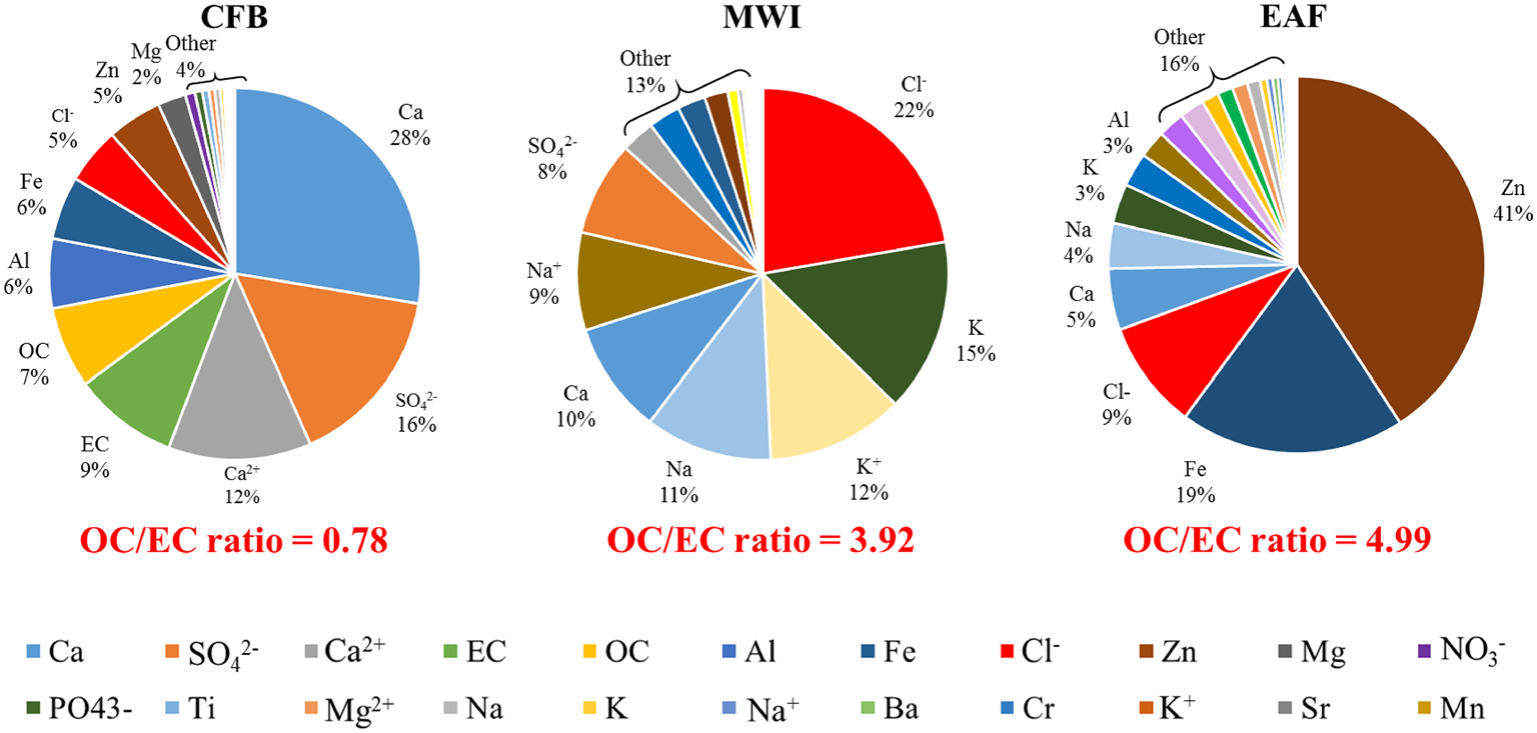
The percentage of chemical species in PM_2.5_ measured in stack gases at different facilities.

The chemical compounds of PM_2.5_ measured in flue gases at different emission sources were shown in Table S1. In CFB, the PM_2.5_ in flue gas had major species of metals as Ca (821,060 ng/m^3^), Al (220,790 ng/m^3^), Fe (171,460 ng/m^3^), the highest water-soluble ions as SO_4_^2−^ (112 ± 29.7 μg/m^3^), and OC/EC ratio as 0.78. In both of MWI and EAF flue gas PM_2.5_, the major species of metals were Ca and Zn, the dominant water-soluble ions were Cl^−^, and OC/EC ratios were greater than 2.0 (Fig. 1). A large OC/EC ratio (> 2.0–2.2) was footprint of secondary organic aerosols^23,24^. It indicated the industrial boiler PM_2.5_ came from primary emitted aerosols. Fig. S1 showed the different contribution of flue gas in PCDD/Fs with CFB, MWI and EAF. Due to the result that ΣPCDD in flue gas was contributed to both phase in CFB and MWI.

### Mass concentration of PM_2.5_, PCDD/Fs levels and chemical compounds in ambient air

In the vicinity of stationary sources, the measurements indicated that the mass concentrations of PM_2.5_ were 10.2 ± 1.71, 12.2 ± 2.08, 10.1 ± 2.65, 11.3 ± 3.73, 29.5 ± 4.29, and 35.1 ± 4.75 μg/m^3^ at site C1, C2, M1, M2, E1, and E2, respectively. The PM_2.5_ mass concentrations measured at downwind sites were higher than upwind sites. In addition, significantly lower PM_2.5_ concentration (4.26 ± 1.59 μg/m^3^) were measured at Mt. Lulin. Concentration of PM_2.5_ at site E2 (downwind site of EAF) was 35.1 ± 4.75 μg/m^3^ and the highest with other sampling sites. All measurements showed that atmospheric PM_2.5_ concentrations were lower than the air quality standards for PM_2.5_ in Taiwan (35 μg/m^3^), except site E2. It may be affected by particulate matter emitted from EAF.

**Figure 2:**
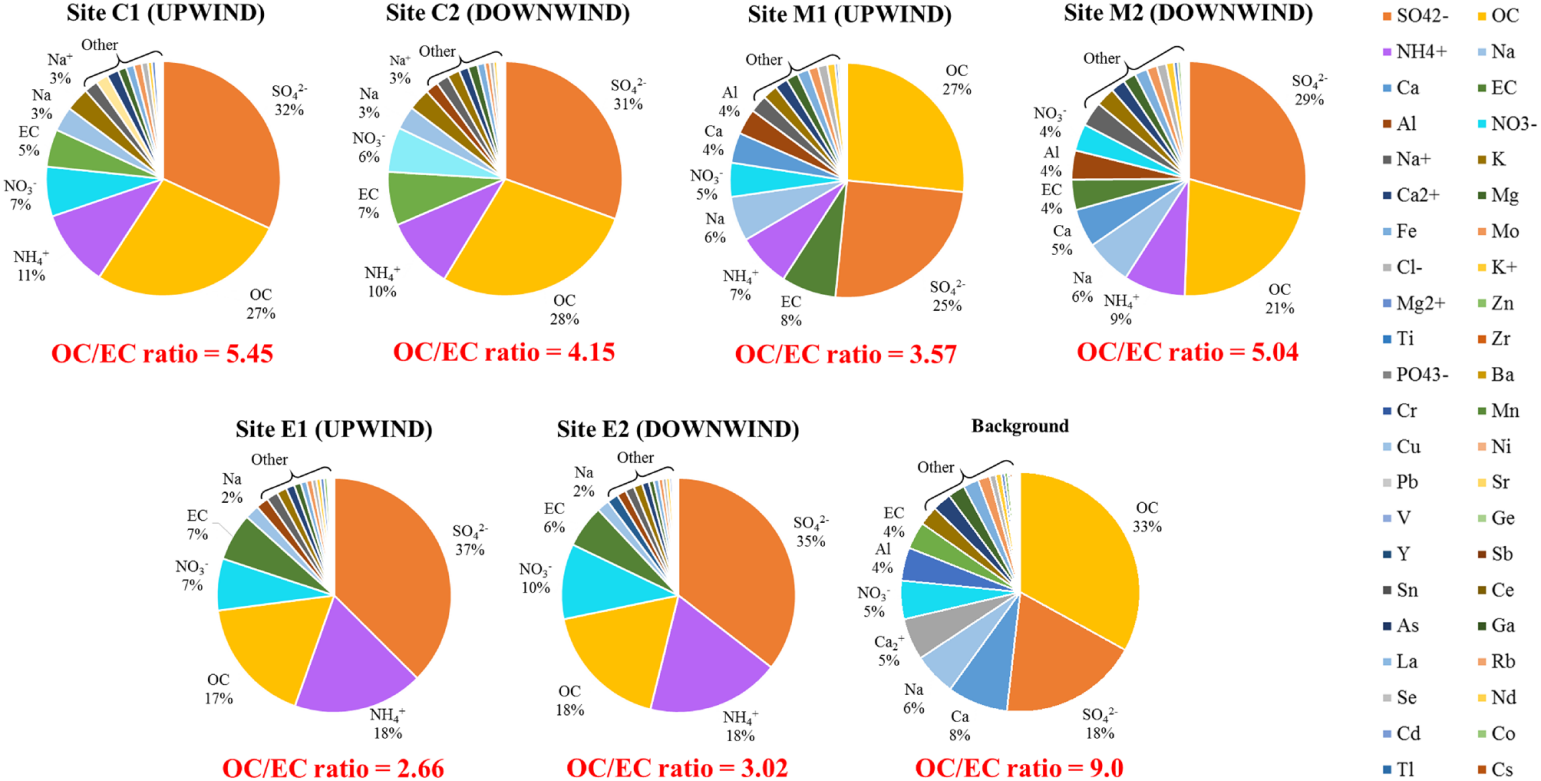
The percentage of atmospheric chemical species in PM_2.5_ in the vicinity of stationary emission sources.

Table 2 shows the atmospheric concentrations of PCDD/Fs in the vicinity of different stationary sources. The highest dioxin concentration (vapor + solid) was 31.1 ± 16.3 fg I-TEQ/m^3^ at site C2 (CFB downwind site), average concentration was 25.5 ± 13.4 and 5.55 ± 2.91 fg I-TEQ/m^3^ in vapor and solid phase, respectively. The results indicated PCDD/Fs had large proportion (82%) in vapor phase and dominant PCDD/Fs species were PCDFs in summer time in this study. Hence, Ngo, et al.^25^ showed the PCDD/Fs concentrations on different season in Taiwan. Obviously, the highest concentration of PCDD/Fs was 27.2 fg I-TEQ/m^3^ in summer in Southern Taiwan. Our result was also similar with the finding of Chi, et al.^26^ that the concentration of PCDD/Fs in vapor phase increased with increasing ambient temperature. The lowest PCDD/Fs concentration was 0.50 ± 0.12 fg I-TEQ/m^3^ at background site (Mt. Lulin), average concentration was 0.18 ± 0.04 and 0.32 ± 0.08 fg I-TEQ/m^3^ in vapor and solid phase, respectively. Fig. S2-S4 also showed the species of PCDD/Fs were contributed in dibenzofurans for vapor phase at vicinity of different stationary sources. Interestingly, Fig. S5 showed the PCDD/F congeners measured in ambient air in the background site was obviously contributed in OCDD both on vapor and solid phase. On the other hand, higher PCDF contributions associated with anthropogenic activities^27^. The Atmospheric chemical compounds of PM_2.5_ at different stations were shown in Supplementary Table S2. For ambient PM_2.5_, Na, K, and Ca was major metals, the water-soluble ions were dominated by SO_4_^2−^. Moreover, we found the ratio between Organic Carbon and Elemental Carbon to be more than 2.0, indicating secondary aerosol origin (Fig. 2).

**Table 2:** Concentrations of atmospheric PM_2.5_, PCDD/Fs and chemical compounds measured in different area.

Site	C 1 (n = 3)	C 2 (n = 3)	M 1 (n = 3)	M 2 (n = 3)	E 1 (n = 3)	E 2(n = 3)	Background (n = 4)
PM_2.5_(μg/m^3^)	10.2 ± 1.71	12.2 ± 2.08	10.1 ± 2.65	11.3 ± 3.73	29.5 ± 4.29	35.1 ± 4.75	4.26 ± 1.59
PCDD/Fs (fg I-TEQ/m^3^)							
PM_2.5_	3.54 ± 2.47	5.55 ± 2.91	1.27 ± 0.724	2.55 ± 2.79	6.34 ± 0.929	8.02 ± 2.15	0.181 ± 0.042
Vapor phase	12.7 ± 6.69	25.5 ± 13.4	2.98 ± 1.71	12.3 ± 8.10	13.0 ± 3.93	21.1 ± 0.760	0.324 ± 0.080
OC(μg/m^3^)	2.40 ± 0.497	2.82 ± 0.519	1.80 ± 1.01	2.55 ± 1.07	4.35 ± 1.14	5.04 ± 1.24	0.995 ± 0.275
EC(μg/m^3^)	0.443 ± 0.312	0.676 ± 0.781	0.358 ± 0.298	0.716 ± 0.563	1.64 ± 0.275	1.67 ± 0.223	0.108 ± 0.017

According to enrichment factor (EF) calculation, concentration for Fe, Mg, Ca, Sr, Ti, Co, and Ce showed low enrichment values (< 1.0) at site C1 and site C2, which coresponded to little influence of anthropogenic flux. Enrichment factor for Na, K, Ba, Mn, Y, Zr, Rb, Cs, Ga, and La were found to be < 10, indicating mixing sources. Significant enrichment (> 10) with Ni, Cu, Zn, Mo, Cd, Sn, Sb, Tl, Pb, V, Cr, As, Se, Nb, and Ge indicating inputs from anthropogenic activities (industrial sources). The metals of PM_2.5_ showed enrichment with Fe, Mg, Sr, Ti, Mn, Co, Rb, and Cs about 1 at site M1 and site M2, indicated they came from crustal elements. Enrichment factor for Na, K, Ca, Ba, and V were found to be < 10, indicating inputs from mixing sources. Significant enrichment with Ni, Cu, Zn, Mo, Cd, Sn, Sb, Tl, Pb, Cr, As, Y, Se, Zr, Ge, Ga, La, Ce, and Nd indicating inputs from industrial sources. The metals of PM_2.5_ showed enrichment with Fe, Mg, Sr, Ti, and Rb about 1 at site E1 and site E2, indicated they came from crustal elements. Enrichment factor for Na, K, Ca, Ba, Mn, Co, Cs, La, Ce and Nd were found to be < 10, indicating inputs from mixing sources. Significant enrichment with Ni, Cu, Zn, Mo, Cd, Sn, Sb, Tl, Pb, Cr, As, Y, Se, Zr, Ge, and Ga indicating inputs from oil sources. Above of the metal composition, they had a little different with the vicinity of stationary sources (Supplementary Table S3).

### Source apportionment of atmospheric PCDD/Fs in PM_2.5_

**Figure 3:**
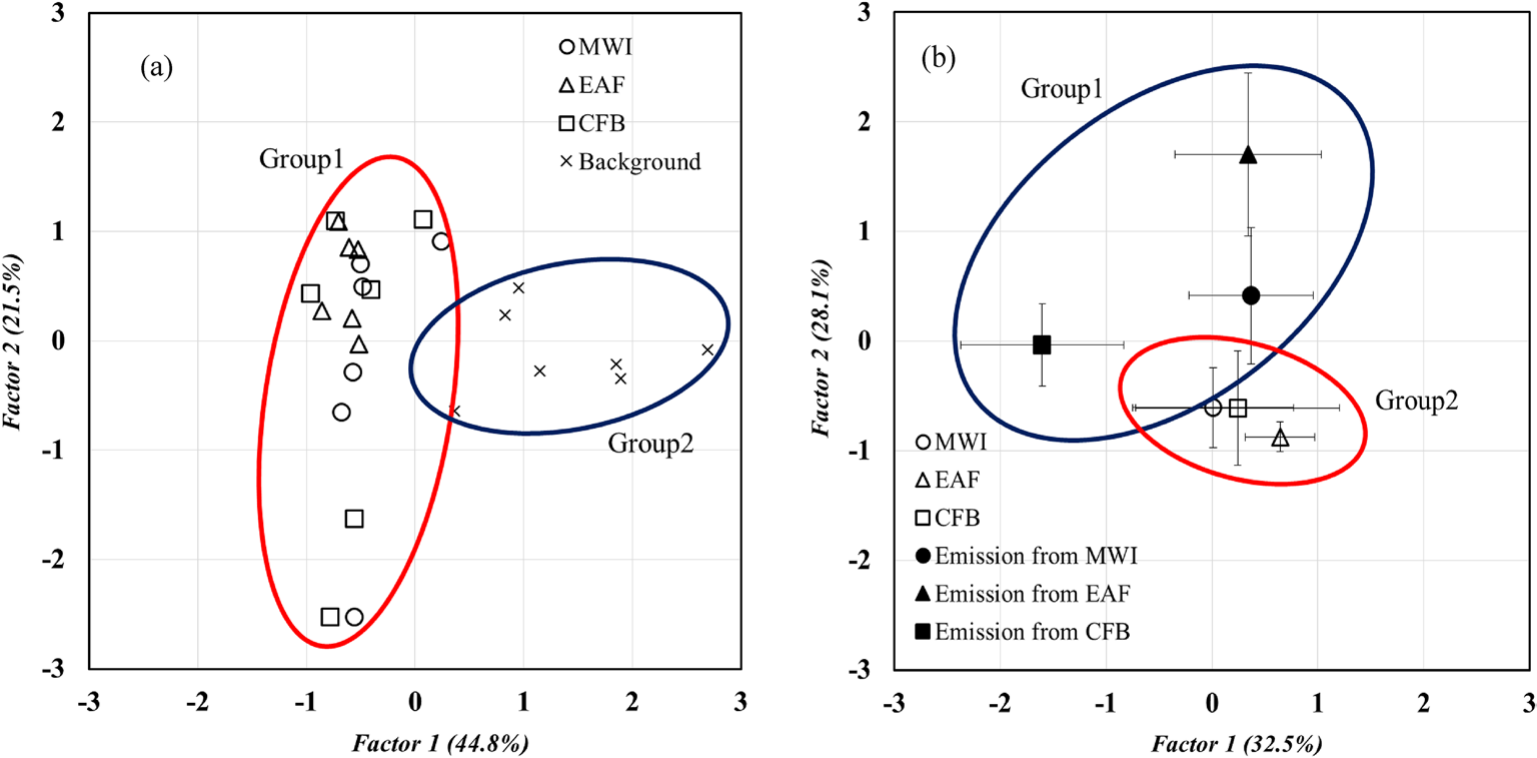
PCA results of atmospheric PCDD/Fs (**a**) in stationary sources vicinity and (**b**) compared with emissions.

According to the principal component analysis (PCA), the Factor 1 and Factor 2 had 44.8%, 21.5% variance in atmospheric PCDD/Fs of PM_2.5_ (Fig. 3a) and 32.5%, 28.1% variance in atmospheric and emission source PCDD/Fs of PM_2.5_ (Fig. 3b). Figure 3a,b also showed the Group 1 and Group 2 were separated to different part. It indicated that Group 1 and Group 2 were formed from different sources. Group 1 and Group 2 were consisted of ambient samples, background samples, respectively in Fig. 3a. On the other hand, Group 1 represented source emission samples when Group 2 was consisted of ambient samples (Fig. 3b). Due to Fig. 3a,b, the result of PCA analysis also showed Group1 and Group2 in ambient, background and emission samples. It also meant that different groups would contribute by different emission sources. However, PCA could not determine the exact source of air pollution the pollution source in detail.

In the next step, the PCDD/Fs congener profile of total twenty-eight air samples were analyzed via PMF model and compared with other study. PMF analysis of atmospheric PCDD/Fs in the vicinity of stationary sources indicated that around 14.6% PCDD/Fs were provided from EAF (correlation coefficient, r = 0.81), 52.6% from coal-fired boiler (correlation coefficient, r = 0.69) and crematoria (correlation coefficient, r = 0.68), 18.0% from traffic activities (correlation coefficient, r = 0.85) and 14.8% from MWIs (correlation coefficient, r = 0.76) (Fig. S6). Highest contributor of PCDD/Fs in this study accounts for combination of coal-fired boiler and crematorium. In Taiwan, many of electricity generating activities relating to coal burning. Coal combustion activities were found to be the largest contributor of PCDD/Fs in Taiwan ambient air (34%) according to Ngo, et al.^25^. On the other hand, there were up to 30 crematoria scattering around Taiwan which also largely contribute to the air pollution on the island^25,28^. Factor 1 (EAF) were dominated to 1,2,3,4,6,7,8-HpCDD and OCDD. Factor 2 (Coal-fired boiler and crematoria) were dominated to 1,2,3,4,6,7,8-HpCDD, OCDD, 1,2,3,4,7,8-HxCDF, 1,2,3,4,6,7,8-HpCDF and OCDF. Compared with the previous study^29^, we used the typical species of PCDD/Fs in traffic emission source to defined factor 3. Factor 3 (Traffic) were dominated to 1,2,3,7,8-PeCDF, 2,3,4,7,8-PeCDF, 1,2,3,4,6,7,8-HpCDF and OCDF. Last, the factor 4 (MWI) were dominated to OCDD, 1,2,3,4,6,7,8-HxCDF, OCDF.

### The mortality relative risk associated with people who exposure PM_2.5_ and PCDD/F

We compared the mortality risk between people living in the highest pollution concentrations with those living in places of lowest air pollution concentrations. For calculating mortality risk of PM_2.5_, we compared the risk between E2 (highest PM_2.5_ concentration) and M1 (lowest PM_2.5_ concentration) sampling areas. On the other hand, C2 (highest PCDD/F concentration) and M1 (lowest PCDD/F concentration) were selected for modelling mortality risk of PCDD/Fs. As the result, there was significant health relative risk for all causes of death, pneumonia, malignant neoplasms, and cancers of intrahepatic bile ducts and liver between the Site E2 with the people who live in the higher mass concentration of PM_2.5_ and the people living in Site M1 in the lower PM_2.5_ concentration. Furthermore, the result presented the significantly higher risk of liver and intrahepatic bile ducts cancers both in male and female at site E2 (Relative risk = 2.427, 95% Confidence Interval = 1.001–5.887, p-value = 0.05). (Table S4).

The people who live in site C2 with a higher concentration of PCDD/F showed the significantly higher relative risk for all causes of death for both males and females than people who live in site M1 PCDD/F (Table S5). The result showed that all people exposure to high-concentration of PCDD/F TEQs were significantly higher health relative risk for all causes of death (Relative risk = 1.236, 95% Confidence Interval = 1.075–1.422, p-value = 0.003). The relative risk of mortality between mass concentrations exposure group in PM_2.5_ and PCDD/Fs were different due to PM_2.5_ combined with other atmospheric components.

Compared with previous study^30^, it was also showed the similar result with the relative risk of mortality between the highest and lowest concentrations of PCDD/Fs and PM_2.5_. Difference of this study was higher correlation with relative risk of mortality in PM_2.5_. The reason was believed that PM_2.5_ contains more hazardous pollutants and leads the difference result with relative risk of mortality in PCDD/Fs and PM_2.5_.

## Discussion

In the  "Mass concentrations of PM_2.5_, PCDD/F levels and chemical compounds in the flue gas of different stationary sources" section, we know the proportion of PCDD/Fs measured in CFB and MWI was different from EAF. The difference can be explained by different air pollution control devices adopted in EAF. The control system in EAF might even generate PCDD/Fs at the temperature window between 200–500 °C via de novo synthesis. In EAF, the flue gas cooling system provides sufficient retention time (2–5 s) with the operating temperature between 300 and 500 °C. On the other hand, ΣPCDF in flue gas was also contributed to both phase in EAF. Previous study^31^ indicates that mostly generates PCDFs in fly ash by the de novo synthesis that was similar with higher PCDFs measured in PM_2.5_ and TPM in the flue gas of EAF. In general, vapor and solid phase distribution of PCDD/F congeners is affected by the temperature variation and removal mechanism in flue gas. Because of the higher vapor pressures of PCDFs compared with PCDDs, the distributions of solid-phase PCDDs in flue gases are higher than that of PCDFs. In EAF, the removal mechanism of solid-phase PCDD/Fs relies on filtration of the bag filter resulting in the increase of PCDF congener distribution observed in stack gas. In addition, the vapor-phase PCDFs in the flue gases of MWI can be effectively removed by the activated carbon injection with bag filter that resulted the lowest PCDF distribution in vapor phase of MWI. Moreover, previous study found that the PCDD/Fs appeared to be present mainly in the solid phase during winter, spring and autumn, while during summer it mostly allocated in gas phase^32^ in the ambient air. All the measurements indicated that the atmospheric PCDD/Fs measured in this study were all lower than the air quality standards for dioxins in Japan (0.6 pg WHO-TEQ/m^3^).

Furthermore, for the limitation of source apportionment, even though the possible sources with the PMF model analysis were given the advice which about sample size (> 100). For the verification of source apportionment in the PMF, we used the previously reported profile of PCDD/Fs from different emission sources to compare with the PCDD/F profile resulted from PMF analysis.

Since PM_2.5_ can serve as holder for PCDD/Fs, the relationship between PM_2.5_ and PCDD/Fs can be represented using the weight of PCDD/Fs on each unit of PM_2.5_ (PCDD/Fs content/PM_2.5_). The PM_2.5_ contents were found at MWI and EAF comparing to that of CFB. In CFB, major burning substances were coal with some level of sulfur. The presence of sulfur might inhibit contents of PCDD/Fs in PM_2.5_ between stack emission and ambient air was listed in Figure S7. Higher the formation of PCDD/Fs^33^. Besides, the burning substances in EAF and MWI were more complex with high contents of chloride (solid waste or wasted steel) which enhance the formation of chloride containing pollution including PCDD/Fs. Therefore, the same amount of PM_2.5_ emitted from difference sources had different content of PCDD/Fs.

Moreover, the contents of PCDD/Fs at downwind sites (C2, M2, E2) were higher than the corresponding upwind sites (C1, M1, E1). Despite the influence of other sources in the vicinity, higher content of PCDD/Fs in PM_2.5_ emitted from the stack did elevate the content of PCDD/Fs in PM_2.5_ in the downwind areas.

## Conclusions

This study was aimed to find the relationship between different emission sources and the vicinity of ambient air with hazardous air pollutants in particulate matters. Especially for the PCDD/Fs distributed in PM_2.5_ were measured from different emission sources. For the dioxin congener distribution, PCDDs were dominant in flue gases of CFB and MWI, PCDFs were dominant in EAF. It may be attributed to the different formation mechanisms among wastes incineration, steel-making, and coal-burning processes. Based on the PCDD/F profiles in flue gases and in the vicinity, the PCDD/F distributions in PM_2.5_ were quite similar in flue gas and the vicinity. As we know, PM_2.5_ is the secondary pollutants in the atmosphere. Hence, the atmospheric PCDD/F concentrations increased with increasing PM_2.5_ at all stations. Moreover, the higher PCDD/F content in PM_2.5_ (227 ± 38.6–460 ± 191 pg I-TEQ/g-PM_2.5_) would be higher in all downwind sampling sites. Ca, Al, and Fe were major metals in CFB flue gas when Ca and Zn dominated in MWI and EAF. In CFB, SO_4_^2−^ was found to be major ion when in MWI and EAF, Cl^−^ was main ion. OC/EC ratio showed primary origin in CFB (OC/EC = 0.78) and secondary origin in MWI and EAF (OC/EC > 2.0).

In the surrounding ambience, the highest level of PM_2.5_ was at site E2 (35.1 ± 4.75 μg/m^3^), the highest dioxin level was at site C2 (31.1 ± 16.3 fg I-TEQ/m^3^). The health relative risk for all causes of death (RR = 1.432, p-value =  < 0.0001) were higher in the high PM_2.5_ exposed group (Site E2). Significant elevation of all-cause mortality risk was observed at high PCDD/F exposed group (RR = 1.236, p-value = 0.003).

## Materials and methods

### Sampling site

In this study, the sampling areas for stationary emission were situated in North and Central Taiwan. The coal-fired boiler (CFB), municipal wastes incinerator (MWI), and electric arc furnace (EAF) were research targets. The flue gas samples were collected for PCDD/Fs and chemical composition analysis from three stationary pollution sources during summer season in 2015. The coal-fired boiler (CFB) locates in Taoyuan city. The boiler (heat recovery system) produced steam and used heat conversion to change the phase of water. The feeding materials of CFB included coal (9.55 ton/hr), waste paper sludge (4.49 ton/hr), and waste tires (4.06 ton/hr), respectively. The steam generator of the boiler system was equipped with flue gas desulfurization system and high-efficiency electrostatic precipitators (dust removal efficiency: 99.0–99.8%). The municipal wastes incinerator (MWI) in this study is located in Taipei city. To control pollution emission, the MWI plant was equipped with dry lime sorbent injection systems coupling with bag filters. Moreover, the control system was enhanced by installation of activated carbon injection technology. The treatment capacity of MWI investigated was 16.6 tons of domestic wastes per hour. The EAF of interest is located in Miaoli city. The capacity of EAF in this study is 70 tons wastes steels per hour and apply bag filters as major control device. The input of the system originated from various sources including wasted building materials, automobile ship scrap iron, industrial scraps, civilian scrap iron and foreign imported scrap iron. Therefore, the concentration of dioxins and heavy metals were normally reported to be relatively high in the flue gas of EAF. Chlorine contents in the flue gas facilitated the re-synthesis of dioxin. In most of the cases, the input fuels are waste plastic, rubber, paint and anti-rust oil, which are contaminated with chlorine, thus could facilitate PCDD/F formation.

**Figure 4:**
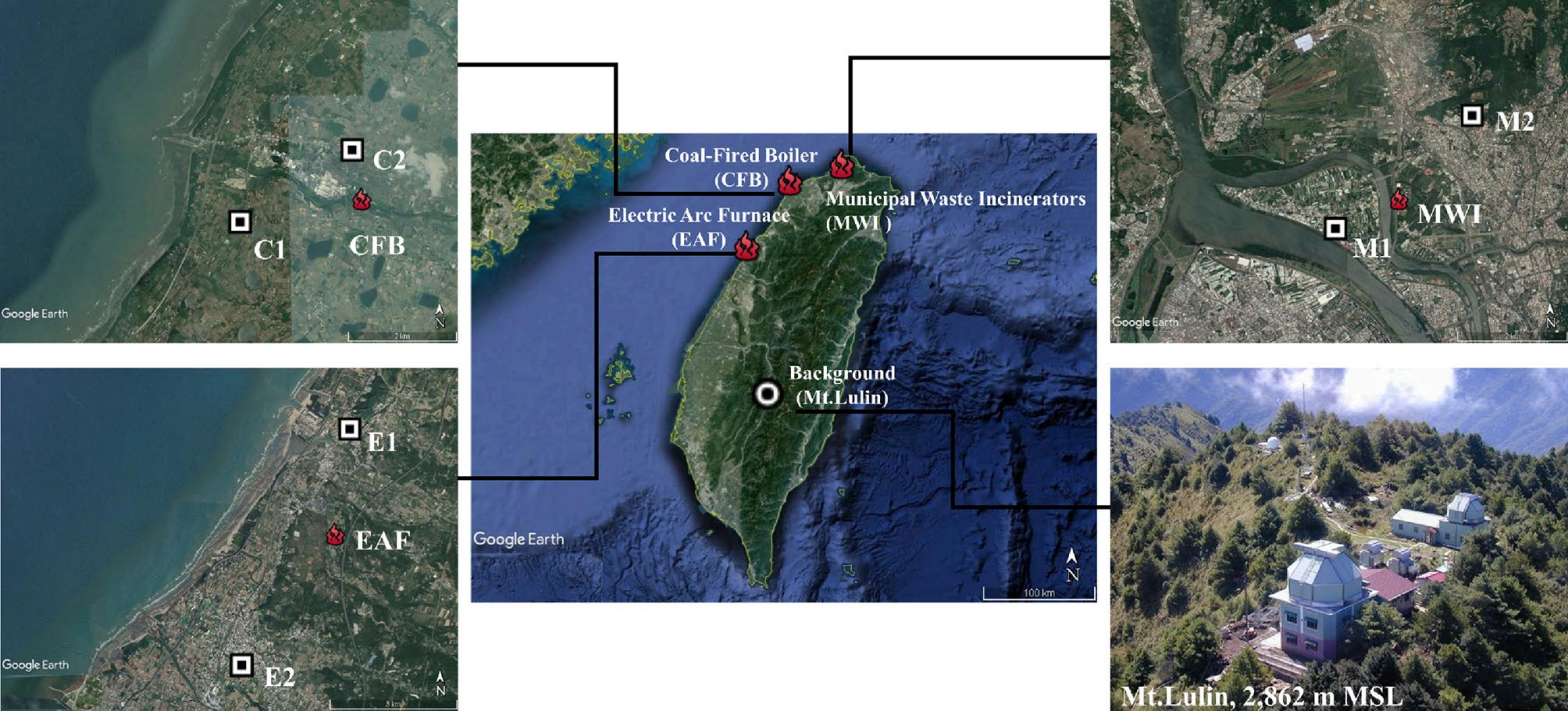
Locations of stationary source and atmospheric PCDD/Fs sampling sites in Taiwan. This map and the picture of Mt.Lulin were provided by the Google earth pro version 7.3.3. and Lulin Observatory (http://www.lulin.ncu.edu.tw/english/), National Central University, respectively.

Additionally, six ambient sampling sites were set up near the locations to the three stationary emission sources. The ambient stations measured PCDD/F and PM_2.5_ concentrations in vapor and solid phases in upwind and downwind sites near the investigated sources. All air samples were taken during spring and summer seasons in 2015. Moreover, a background concentration of PCDD/Fs in Taiwan was selected based on the meteorological information in the high-altitude sampling sites in Central Taiwan (Fig. 4). During CFB flue gas sampling periods (2015/6/12-18), the prevalent winds in the area came from southwest. The ambient sampling site C1 (upwind) and C2 (downwind) were located about 3 km from the coal-fired boiler. The upwind and downwind sampling sites were located at empty space surrounded by farmland, factories, and residents respectively. During MWI sampling periods (2015/8/15-20), prevail winds in the area were southwest. The ambient sampling site M1 (upwind) and M2 (downwind) were located about 3 km from the plant. The upwind and downwind sampling sites were located at the residential area close to incinerator pollution source, complex type potential sources by small factories in pollution areas, respectively. During EAF sampling periods (2015/9/19–24), the dominant winds in the area came from northeast. The ambient sampling site E1 (upwind) and E2 (downwind) were located about 8 km from the plant. The upwind and downwind sampling sites were located nearby power plant and farmland, respectively. On the other hand, the background station was located at Mt. Lulin (23.51–°N, 120.92–°E; 2,862 m above mean sea level) in Jade Mountain National Park. Its high elevation kept it away from all local pollution sources.

### Sampling method

The sampling procedures of stack gases of different facilities were performed following the main guideline of the Taiwan EPA NIEA A212.10B for flue gas collection^34^. The vapor-phase PCDD/Fs in flue gas was collected via XAD-2 while the PM_2.5_ and total particulate matter (TPM) was collected by the cyclone splitter with quartz fiber filter. Isokinetic sampling was ensured to collect representative samples. For flue gas sampling, one TPM sample and five PM_2.5_ samples were collected in CFB; in MWI and EAF, three TPM and three PM_2.5_ samples were collected in each stack.

Additionally, three ambient air samples were collected at each upwind and downwind site of CFB, MWI and EAF, respectively. For ambient air samples, both vapor phase and solid phase (PM_2.5_) samples of PCDD/F compounds were collected using high volume sampling instruments (Analitica HVS-PM_2.5_) and at flow rate of 500L min^−1^. The air sample with total volume was over 700 m^3^ for a typical 24-h sampling duration. Whatman quartz fiber filters and polyurethane foam (PUF) plugs were used for collecting particles and vapor PCDD/F compounds, respectively. The filters were heated at 900 °C (5 h). Gravimetric analysis was done after stabilizing the filter at constant humidity (45% ± 5%) and temperature (18 °C) for at least 24 h. On the other hand, polyurethane foam (PUF) was cleaned using Toluene by Soxhlet purification for 4 h.

### Chemical analysis

In this study, the congeners of seventeen 2,3,7,8-substituted PCDD/F were analyzed with high-resolution gas chromatography (TRACE GC, Thermo Fisher Scientific, USA)/high-resolution mass spectrometry (HRMS) (DFS, Thermo Fisher Scientific, USA) equipped with column DM-5 MS (fused silica capillary, Length: 60 m, I.D.: 0.25 mm, Film: 0.25 μm, DiKMA). After sampling, all filter samples were conditioned similar to pre-sampling condition (humidity of 45% ± 5% and temperature of 18 °C) before weighing. Ion component analysis and metal analysis used up one eighth of the filter, each. Half of each filter went to PCDD/F analysis. One quarter of filter was used as backup.

After Soxhlet extraction and purification, high-resolution gas chromatograph/mass spectrometer was used for PCDD/Fs analysis. The detailed protocol can be found elsewhere^35^. A laboratory blank and filed blank were analyzed for quality control. Furthermore, a matrix spike sample (2.0–20 pg µL^−1^ PCDD/Fs) also were analyzed after every eight samples. The injection volume was 1 µL and the sample volume was 1 mL.

For metal analysis, filters were first digested by acid mixture of HNO_3_/HF (4 ml/2 ml) coupling with microwave digestion system (MARSX press, CEM Corporation, Matthews, NC, USA). Inductively coupled plasma optical emission spectrometry (ICP-OES) (Optima 2100DV, PerkinElmer Instruments, USA) was used for metal analysis. One eighth of filter for ion analysis was sonicated for 90 min. The following compound ion Cl^−^, NO_3_^−^, SO_4_^2−^, PO_4_^3−^, Na^+^, NH_4_^+^, K^+^, Ca^2+^, and Mg^2+^ were analyzed using ion chromatograph (IC).

### Enrichment factor

In order to evaluate the enrichment of each element relative to the crust composition, this study calculated the enrichment factor (EF) for each element, which was calculated by Eq. (1).

1 $${\text{EF}} = \left( {\text{E/Al}} \right)\,{\text{Sample/}}\,\left( {\text{E/Al}} \right)\,{\text{Crust}}$$

where E is the enrichment value of the element relative to the source of crust (Al), (E/Al) Sample is the ratio that element E to the content of Al in the sample, (E/Al) Crust is the ratio that element E to the content of Al in average composition of crust.

EF value equal to 1.0 means that the element is mainly from the source of crust. When the EF value is more than 10, it means that the element mainly come from other sources of anthropogenic pollution. When the EF value ranges from 2.0 to 10, it means that the element might have a mixed source of crust and anthropogenic pollution.

### Source apportionment

To identify the sources of the atmosphere PM_2.5_, principal component analysis (PCA) and the Positive Matrix Factorization (PMF, version 5.0) which available from U.S. EPA (2014) were used to identify and quantify sources that contribute to ambient PCDD/F concentrations in the vicinity of stationary pollution sources.

We used PCA to reduce the dimension of original PCDD/Fs into different major principal components with different loading scores. On the other hand, PMF was used to decompose PCDD/Fs into different factors. PMF can result in PCDD/F fingerprints of different factors. These fingerprints can be used to compare with other known fingerprint from emission sources to identify the possible sources of PCDD/Fs.

### Relative risk of mortality in statistical analysis

Assuming the proportion of PCDD/Fs in each gram of PM_2.5_ is constant, we estimated the concentrations of PCDD/Fs in the sampling areas from PM_2.5_ concentration collected by automatic monitoring system (Taiwan EPA) and the coefficient of PCDD/Fs content in PM_2.5_ from our research (Formula 2).

2 $$Concentration = A \times B \times C$$

Concentration: PCDD/F concentrations (fg I-TEQ/m^3^); A: Content of PCDD/Fs in PM_2.5_ (pg I-TEQ/g-PM_2.5_); B: PM_2.5_ ratio between manual and automatic sampling systems; C: PM_2.5_ automatic monitoring concentrations (μg/m^3^).

We separately compared the risk of mortality by exposing to PM_2.5_ and PCDD/Fs. The comparing groups are places with the highest concentration (C2: downwind site of CFB for PCDD/Fs; E2: downwind site of EAF for PM_2.5_) and places with lowest concentration (M1: upwind site of MWI for both PCDD/Fs and PM_2.5_). The mortality information at township level was achieved from the National Mortality Registry data, Taiwan Ministry of Health and Welfare. The association was modeled by Poisson regression model using SAS 9.4.

## Supplementary Information

Supplementary information.

## Data Availability

The data with cause of death during the current study was available from Ministry of Health and Welfare: https://www.mohw.gov.tw/np-128-2.html.
