# Supplementary material for: Characteristics of PCDD/Fs in PM2.5 from emission stacks and the nearby ambient air in Taiwan
Source: Sci Rep. 2021 Apr 14;11:8093. doi: 10.1038/s41598-021-87468-5 (PMC8046994; doi:10.1038/s41598-021-87468-5)
Supplement: Supplementary file 1 — Supplementary information. [file 41598_2021_87468_MOESM1_ESM.docx]

**Characteristics of PCDD/Fs in PM_2.5_ from Emission Stacks and the Nearby Ambient Air in Taiwan**

Supplementary Material

**Shih Yu Pan ^1,2^, Yi Ting Liou^1^, Moo Been Chang ^3^, Charles C.-K. Chou ^4^,**

**Tuan Hung Ngo^2, 5^ and Kai Hsien Chi ^1,2^***

^1^ Institute of Environmental and Occupational Health Sciences, National Yang Ming Chiao Tung University, Taipei 112, Taiwan.

^2^ College of medicine, National Yang Ming Chiao Tung University, Hsinchu 300, Taiwan.

^3^ Graduate Institute of Environmental Engineering, National Central University, Chungli 320, Taiwan.

^4^ Research Center for Environmental Changes, Academia Sinica, Taipei 115, Taiwan.

^5^ International Health Program, National Yang Ming Chiao Tung University, Taipei, 112, Taiwan.

Taiwan.

**Corresponding author:**

Kai Hsien Chi, email: khchi2@ym.edu.tw

**First author:**

Shih Yu Pan, email: shelly.pan923@hotmail.com

**Co-author:**

Yi Ting Liou, email: yiti0408@gmail.com

Moo Been Chang, email: mbchang@ncuen.ncu.edu.tw

Charles C.-K. Chou, email: ckchou@gate.sinica.edu.tw

Tuan Hung Ngo, email: tuanhung0712@gmail.com

Table S1 Chemical compounds of PM_2.5_ measured in flue gas of different facilities

| **Emission Sources (μg/m^3^)** | **CFB (n=6)** | **MWI (n=6)** | **EAF (n=6)** |
| --- | --- | --- | --- |
| **Na^+^** | 1.16±0.27 | 36.4±7.78 | 7.69±2.66 |
| **NH_4_^+^** | 0.185 | - | 0.206±0.183 |
| **K^+^** | 0.641±0.099 | 51.0±11.9 | 7.42±2.42 |
| **Mg^2+^** | 3.67±1.71 | 0.436±0.121 | 0.386±0.318 |
| **Ca^2+^** | 88.3±16.5 | 12.5±4.56 | 1.96±0.567 |
| **Cl^-^** | 34.9±55.2 | 95.1±25.0 | 30.3±9.67 |
| **NO_3_^-^** | 5.96±5.19 | - | 4.32±1.52 |
| **PO_4_^3-^** | 4.51±0.906 | 0.468±0.069 | - |
| **SO_4_^2-^** | 112±29.7 | 35.2±9.01 | 3.56±0.822 |

Table S2 Concentrations of water-soluble ion in atmospheric PM_2.5_ measured in the vicinity of different emission sources.

| **Site**  **(μg/m^3^)** | **C 1**  **(n=3)** | **C2**  **(n=3)** | **M 1**  **(n=3)** | **M 2**  **(n=3)** | **E 1**  **(n=3)** | **E 2**  **(n=3)** | **Background**  **(n=4)** |
| --- | --- | --- | --- | --- | --- | --- | --- |
| **Na^+^** | 0.286±0.096 | 0.305±0.081 | 0.284±0.298 | 0.224±0.256 | 0.188±0.027 | 0.191±0.031 | 0.024±0.015 |
| **NH_4_^+^** | 0.936±0.380 | 0.990±0.400 | 0.684±0.573 | 0.631±0.655 | 4.47±0.504 | 5.21±0.599 | 0.079±0.068 |
| **K^+^** | 0.150±0.025 | 0.175±0.038 | 0.091±0.025 | 0.103±0.029 | 0.286±0.123 | 0.337±0.113 | 0.013±0.008 |
| **Mg^2+^** | 0.043±0.011 | 0.044±0.011 | 0.046±0.030 | 0.036±0.026 | 0.049±0.009 | 0.054±0.014 | 0.023±0.007 |
| **Ca^2+^** | 0.144±0.013 | 0.170±0.014 | 0.170±0.033 | 0.173±0.035 | 0.149±0.018 | 0.163±0.027 | 0.169±0.032 |
| **Cl^-^** | 0.095±0.099 | 0.066±0.038 | 0.105±0.422 | 0.130±0.339 | 0.235±0.044 | 0.266±0.069 | 0.048±0.019 |
| **NO_3_^-^** | 0.606±0.059 | 0.619±0.101 | 0.287±0.160 | 0.422±0.266 | 1.69±0.827 | 2.87±0.928 | 0.156±0.080 |
| **PO_4_^3-^** | 0.017±0.010 | 0.025±0.010 | 014±0.005 | 0.017±0.006 | 056±0.026 | 0.058±0.029 | 005±0.002 |
| **nss-SO_4_^2-^** | 2.74±0.838 | 2.99±0.922 | 2.33±1.56 | 2.18±1.65 | 9.29±0.843 | 10.0±0.599 | 0.557±0.236 |

Table S3 Enrichment Factor (EF) value of trace metals in atmospheric PM_2.5_ measured in the vicinity of different emission sources.

| **Site**  **Compound** | | **C1** | **C2** | **M1** | **M2** | **E1** | **E2** | **Background** |
| --- | --- | --- | --- | --- | --- | --- | --- | --- |
| **Aluminum** | **Al** | 1.0 | 1.0 | 1.0 | 1.0 | 1.0 | 1.0 | 1.0 |
| **Ferrum Iron** | **Fe** | 1.58 | 1.5 | 0.55 | 0.63 | 0.83 | 0.87 | 0.5 |
| **Sodium** | **Na** | 11.6 | 8.89 | 6.01 | 5.46 | 4.83 | 5.00 | 2.25 |
| **Magnesium** | **Mg** | 1.87 | 1.59 | 1.64 | 1.55 | 1.38 | 1.45 | 1.16 |
| **Potassium** | **K** | 6.88 | 5.44 | 2.28 | 2.59 | 3.97 | 4.55 | 1.51 |
| **Calcium** | **Ca** | 1.64 | 1.63 | 2.33 | 2.57 | 2.43 | 2.48 | 2.96 |
| **Strontium** | **Sr** | 2.06 | 1.39 | 1.46 | 1.48 | 1.84 | 2.03 | 1.35 |
| **Barium** | **Ba** | 5.73 | 3.18 | 4.93 | 6.00 | 5.7 | 6.65 | 7.41 |
| **Titanium** | **Ti** | 0.7 | 0.79 | 0.55 | 0.68 | 0.61 | 0.68 | 0.6 |
| **Manganese** | **Mn** | 3.54 | 5.57 | 1.31 | 1.58 | 3.57 | 3.25 | 1.14 |
| **Cobalt** | **Co** | 1.49 | 2.08 | 1.24 | 1.3 | 3.17 | 2.34 | 0.88 |
| **Nickel** | **Ni** | 23.6 | 18.3 | 12.5 | 12.4 | 32.8 | 21.2 | 24.9 |
| **Copper** | **Cu** | 56.9 | 250 | 17.4 | 21.2 | 44.1 | 52.5 | 18.8 |
| **Zinc** | **Zn** | 283 | 218 | 100 | 114 | 303 | 371 | 51 |
| **Molybdenum** | **Mo** | 9114 | 4,499 | 20289 | 19516 | 19759 | 20563 | 3839 |
| **Cadmium** | **Cd** | 1130 | 875 | 252 | 317 | 1076 | 1123 | 122 |
| **Tin** | **Sn** | 1631 | 1020 | 123 | 168 | 503 | 519 | 21 |
| **Antimony** | **Sb** | 3331 | 3328 | 1251 | 2013 | 2725 | 3422 | 143 |
| **Thallium** | **Tl** | 62.1 | 69.5 | 13.6 | 19.4 | 59.2 | 65 | 6.25 |
| **Lead** | **Pb** | 270 | 279 | 91.6 | 70.3 | 228 | 251 | 22.6 |
| **Vanadium** | **V** | 40.3 | 24.6 | 3.95 | 3.86 | 15.4 | 16.9 | 1.71 |
| **Chromium** | **Cr** | 21.9 | 17 | 17.6 | 19.6 | 23.1 | 21.8 | 20.8 |
| **Arsenic** | **As** | 254 | 215 | 61.4 | 84.9 | 282 | 310 | 14.4 |
| **Yttrium** | **Y** | 6.66 | 3.53 | 14.1 | 13.6 | 13.9 | 14.7 | 12.5 |
| **Selenium** | **Se** | 28137 | 18982 | 1180 | 1998 | 9572 | 10803 | 1509 |
| **Zirconium** | **Zr** | 11.6 | 7.00 | 20.9 | 20.8 | 21.3 | 22.5 | 17.4 |
| **Germanium** | **Ge** | 176 | 127 | 359 | 357 | 366 | 380 | 267 |
| **Rubidium** | **Rb** | 2.62 | 2.43 | 1.09 | 1.22 | 1.87 | 2.11 | 1.08 |
| **Caesium** | **Cs** | 4.08 | 4.32 | 1.65 | 2.00 | 4.58 | 5.13 | 1.46 |
| **Gallium** | **Ga** | 10.9 | 7.88 | 8.4 | 9.28 | 12.8 | 14.7 | 9.79 |
| **Lanthanum** | **La** | 2.92 | 3.01 | 3.76 | 3.77 | 3.83 | 3.97 | 2.37 |
| **Cerium** | **Ce** | 1.38 | 1.5 | 3.16 | 3.26 | 3.13 | 3.24 | 2.35 |
| **Neodymium** | **Nd** | 1.74 | 1.7 | 3.4 | 3.52 | 3.37 | 3.5 | 2.5 |

Table S4 The relative risk of mortality between PM_2.5_ mass concentrations exposure group in E 2 vs M 1.

| **E 2 *vs* M 1**  Cause of Death | ALL | | Male | | Female | |
| --- | --- | --- | --- | --- | --- | --- |
|  | Relative Risk(95%CI) | p-value | Relative Risk(95%CI) | p-value | Relative Risk(95%CI) | p-value |
| All causes of death (A00-Y98) | 1.432(1.251-1.640) | ***<.0001*** | 1.384(1.229-1.557) | ***<.0001*** | 1.492(1.272-1.750) | ***<.0001*** |
| Cerebrovascular disease (I60-I69) | 1.473(0.863-2.513) | 0.156 | 1.327(0.836-2.105) | 0.230 | 1.589(0.851-2.968) | 0.146 |
| Pneumonia (J12-J18) | 1.992(1.146-3.462) | ***0.015*** | 2.084(1.296-3.349) | ***0.002*** | 1.777(0.910-3.471) | 0.092 |
| Malignant neoplasms (C00-C97) | 1.362(1.065-1.741) | ***0.014*** | 1.408(1.135-1.746) | ***0.002*** | 1.273(0.955-1.696) | 0.100 |
| Cancers of liver and intrahepatic bile ducts (C22) | 2.158(1.125-4.139) | ***0.021*** | 2.005(1.182-3.403) | ***0.01*** | 2.427(1.001-5.887) | ***0.050*** |
| Cancers of trachea, bronchus and lung (C33-C34) | 1.432(0.808-2.536) | 0.219 | 1.297(0.774-2.174) | 0.323 | 1.596(0.842-3.027) | 0.152 |
| Cancers of colon, rectum and anus (C18-C21) | 1.154(0.576-2.309) | 0.686 | 0.951(0.490-1.845) | 0.881 | 1.404(0.678-2.904) | 0.361 |
| Female breast cancer (C50) | - | - | - | - | 1.349(0.595-3.057) | 0.474 |

Table S5. The relative risk of mortality between PCDD/F concentrations exposure group in C2 vs M1.

| **C 2 *vs* M 1**  Cause of Death | ALL | | Male | | Female | |
| --- | --- | --- | --- | --- | --- | --- |
|  | Relative Risk(95%CI) | p-value | Relative Risk(95%CI) | p-value | Relative Risk(95%CI) | p-value |
| All causes of death (A00-Y98) | 1.236(1.075-1.422) | ***0.003*** | 1.158(1.024-1.309) | ***0.020*** | 1.276(1.082-1.505) | ***0.004*** |
| Cerebrovascular disease (I60-I69) | 1.521(0.895-2.587) | 0.121 | 1.344(0.848-2.129) | 0.209 | 1.526(0.813-2.864) | 0.188 |
| Pneumonia (J12-J18) | 0.987(0.520-1.872) | 0.968 | 0.843(0.474-1.501) | 0.563 | 1.101(0.525-2.307) | 0.800 |
| Malignant neoplasms (C00-C97) | 1.094(0.845-1.416) | 0.496 | 1.053(0.837-1.326) | 0.659 | 1.099(0.817-1.479) | 0.532 |
| Cancers of liver and intrahepatic bile ducts (C22) | 1.466(0.729-2.947) | 0.283 | 1.121(0.619-2.031) | 0.706 | 2.121(0.858-5.240) | 0.103 |
| Cancers of trachea, bronchus and lung (C33-C34) | 1.275(0.710-2.291) | 0.416 | 1.363(0.818-2.273) | 0.235 | 1.046(0.519-2.111) | 0.900 |
| Cancers of colon, rectum and anus (C18-C21) | 0.965(0.468-1.992) | 0.923 | 0.922(0.473-1.800) | 0.813 | 0.950(0.429-2.105) | 0.899 |
| Female breast cancer (C50) | - | - | - | - | 1.096(0.465-2.583) | 0.834 |


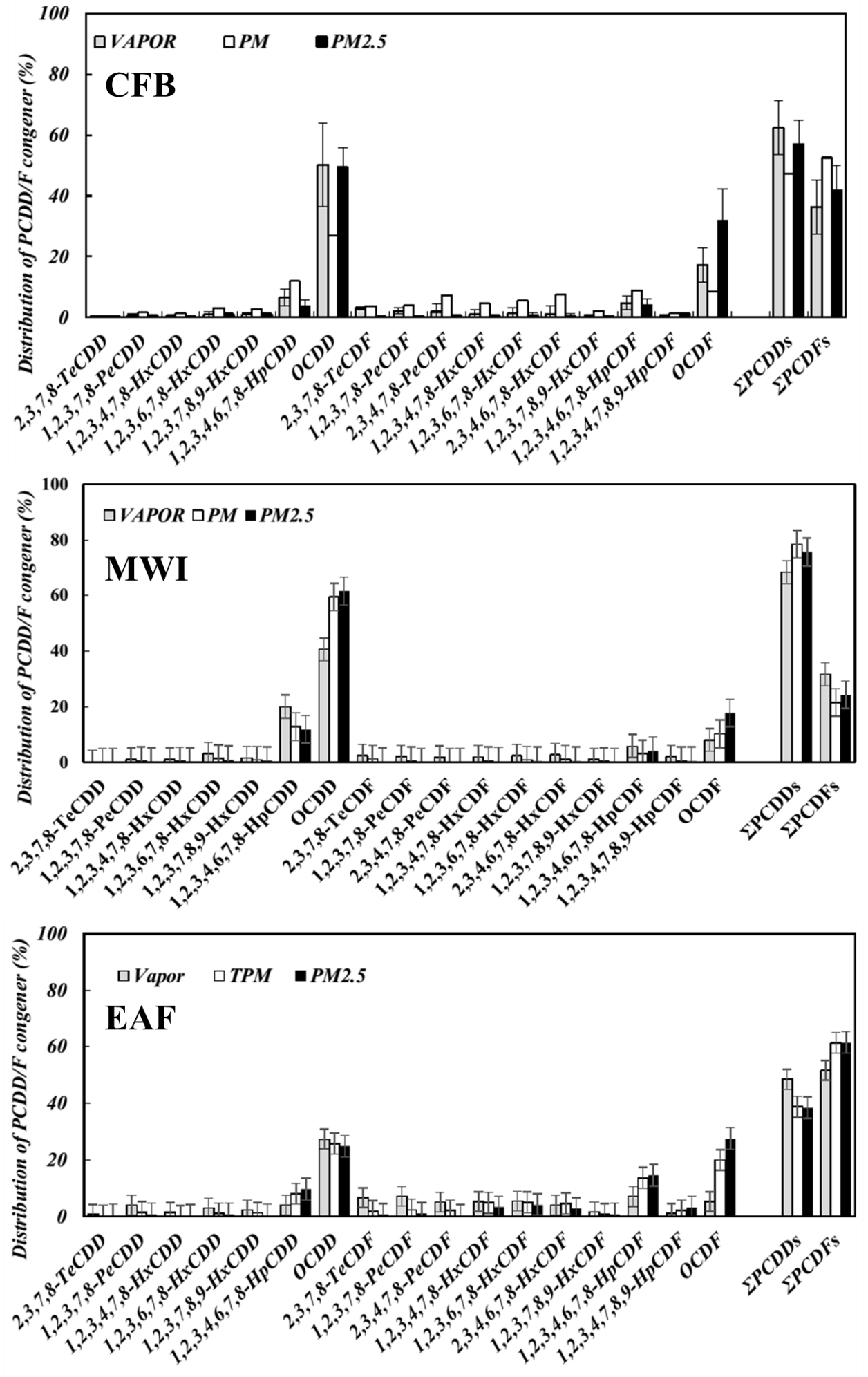


Fig.S1 The distribution of PCDD/F congeners measured in stack gases at different facilities.


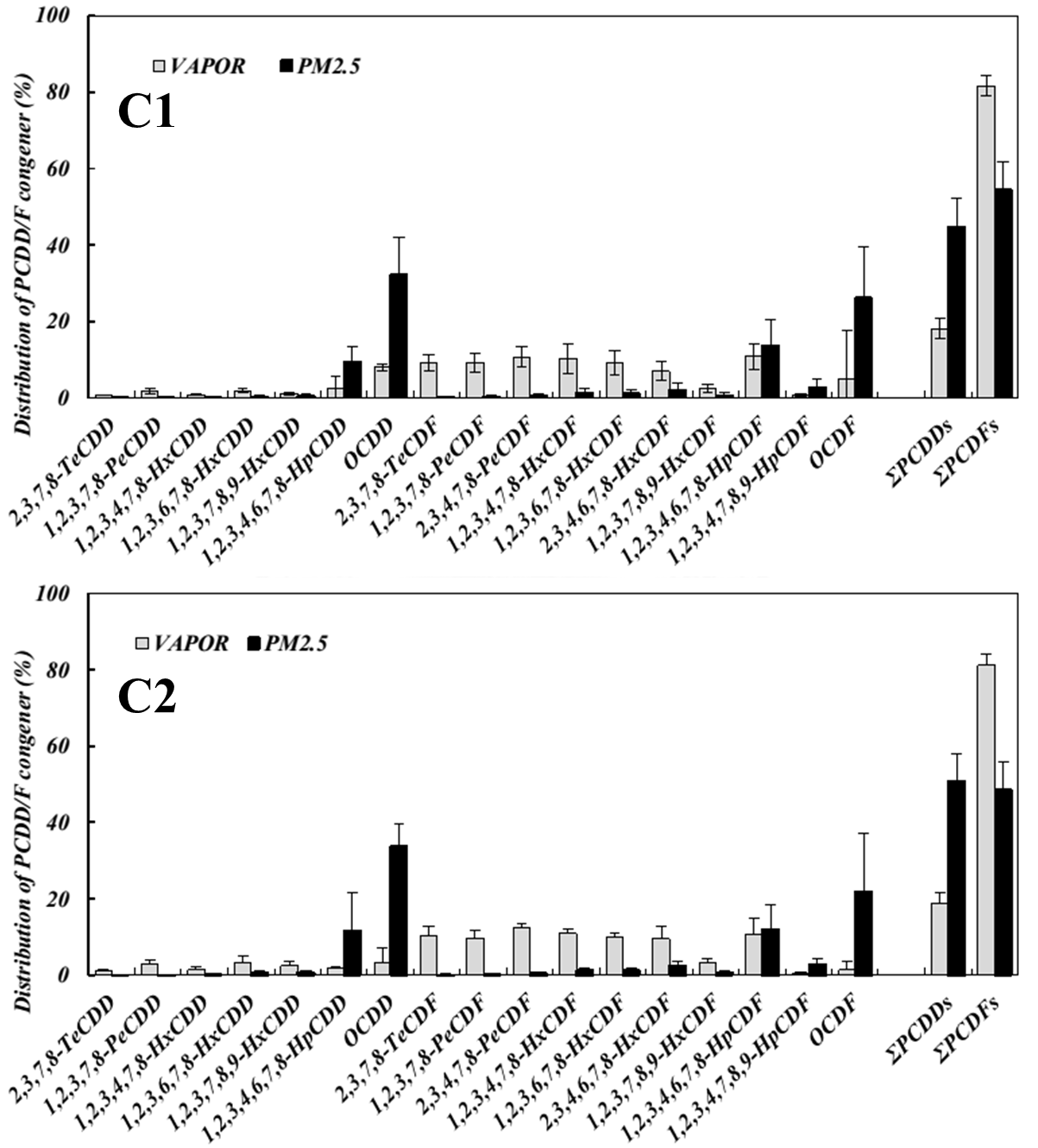


Fig.S2 The distribution of PCDD/F congeners measured in ambient air in the coal-fired boiler (CFB).


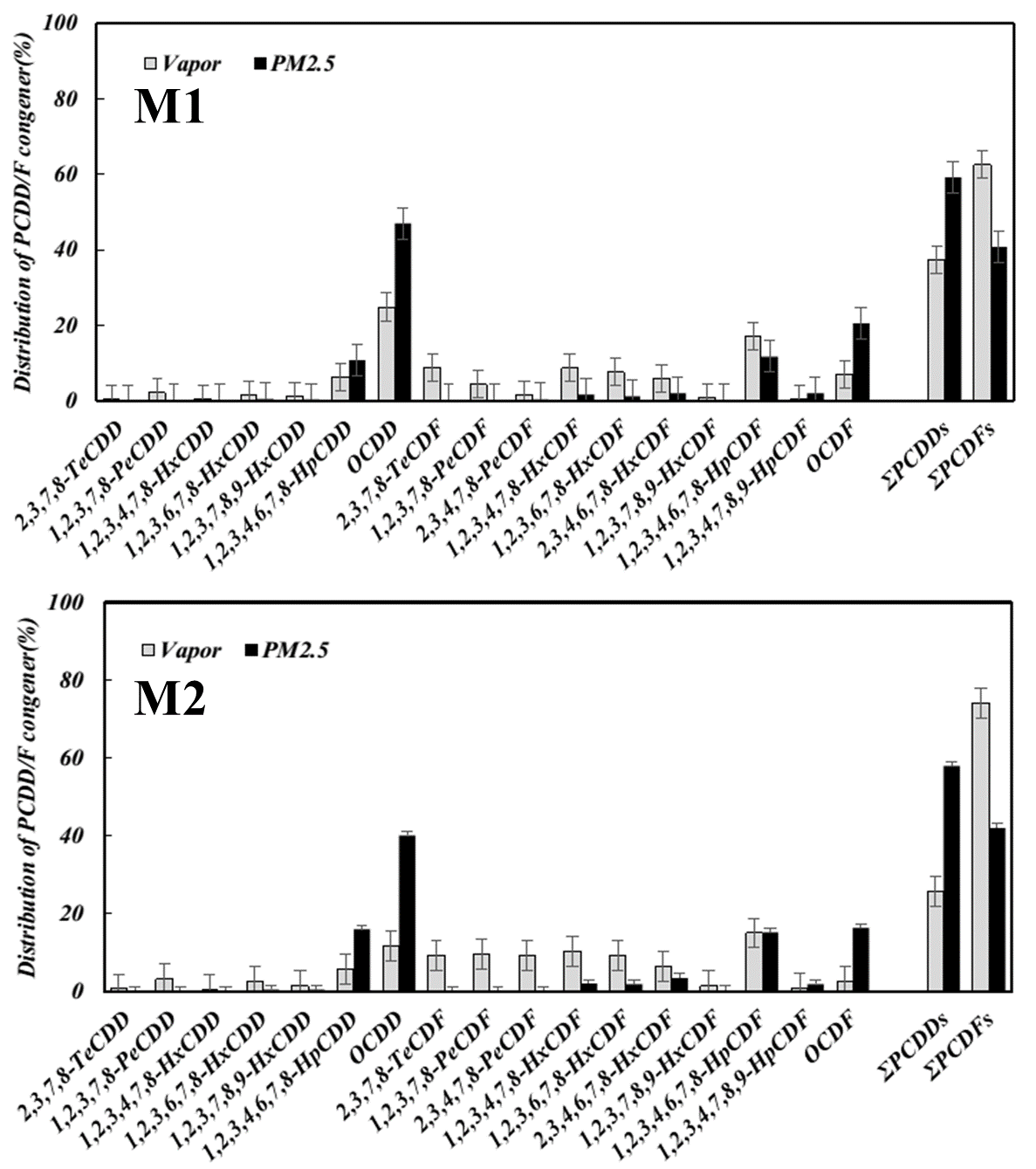


Fig.S3 The distribution of PCDD/F congeners measured in ambient air in the municipal waste incinerator (MWI).


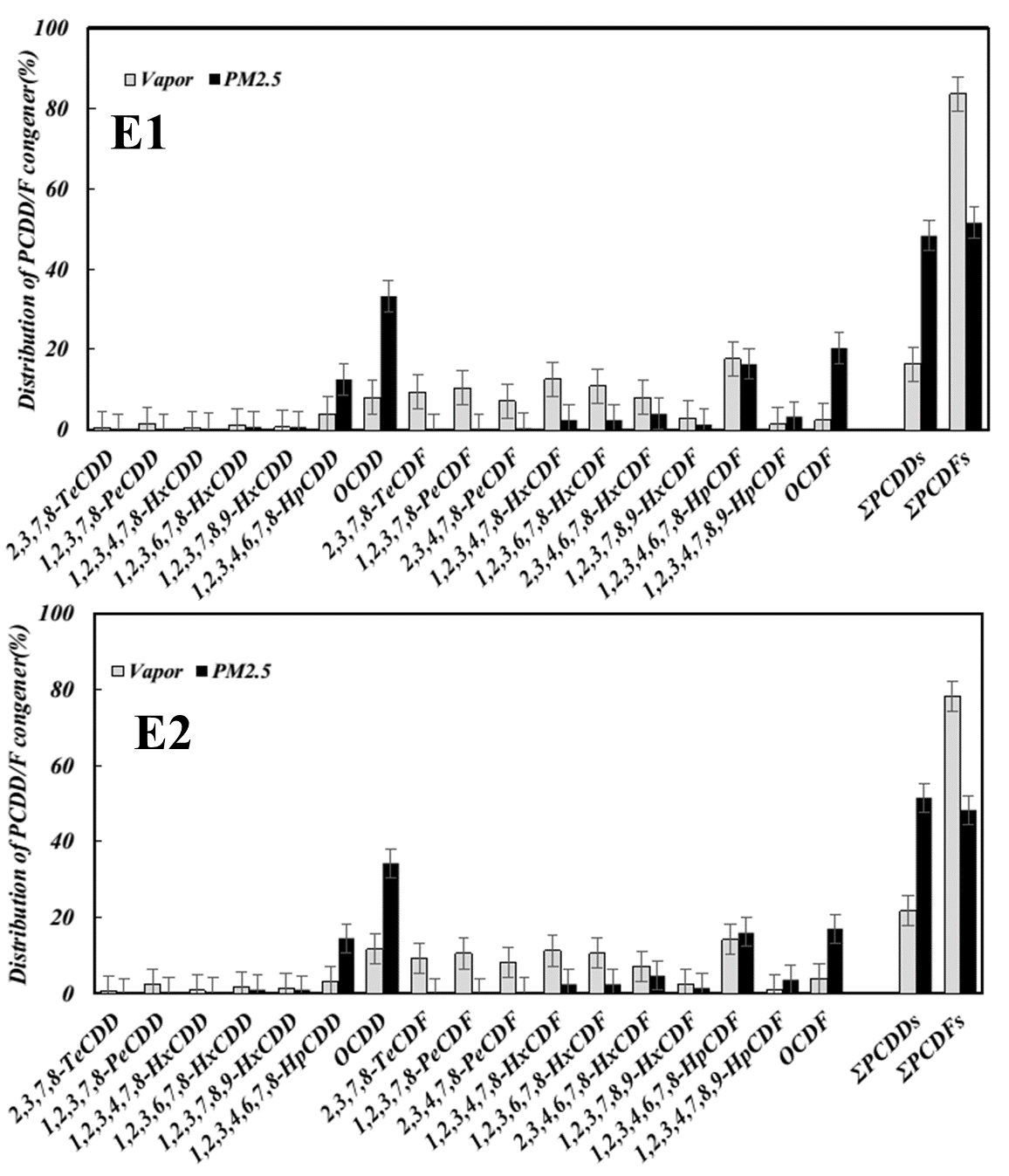


Fig.S4 The distribution of PCDD/F congeners measured in ambient air in the electric arc furnace (EAF).


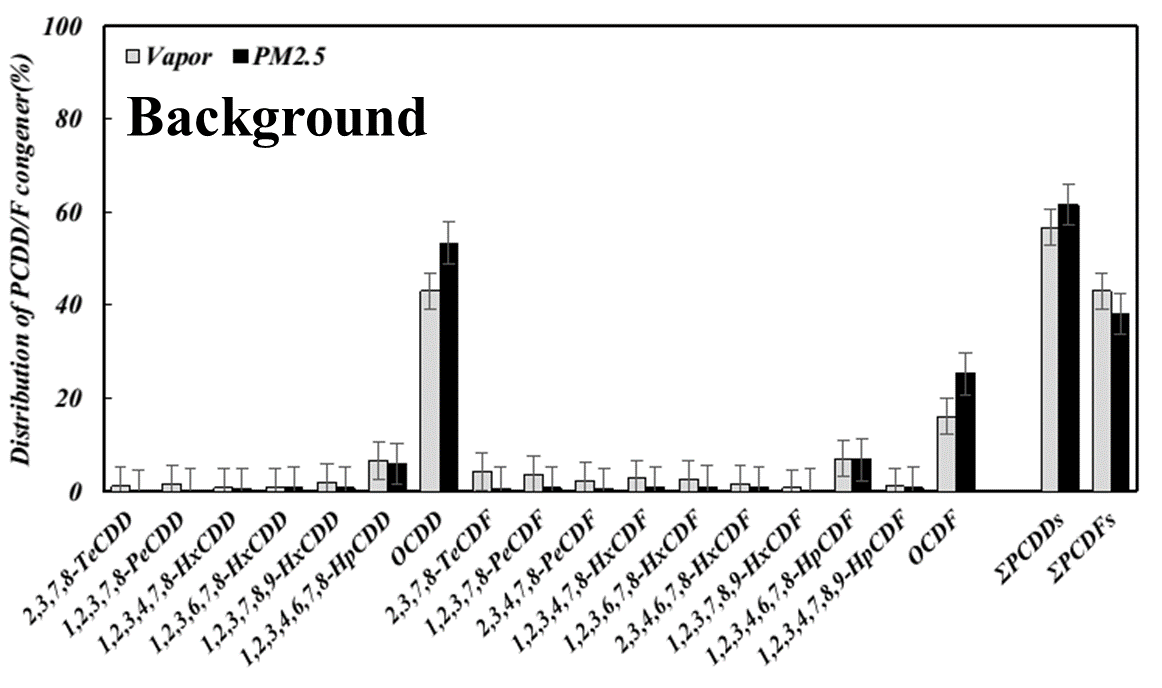


Fig.S5 The distribution of PCDD/F congeners measured in ambient air in the background site (Mt.Lulin).


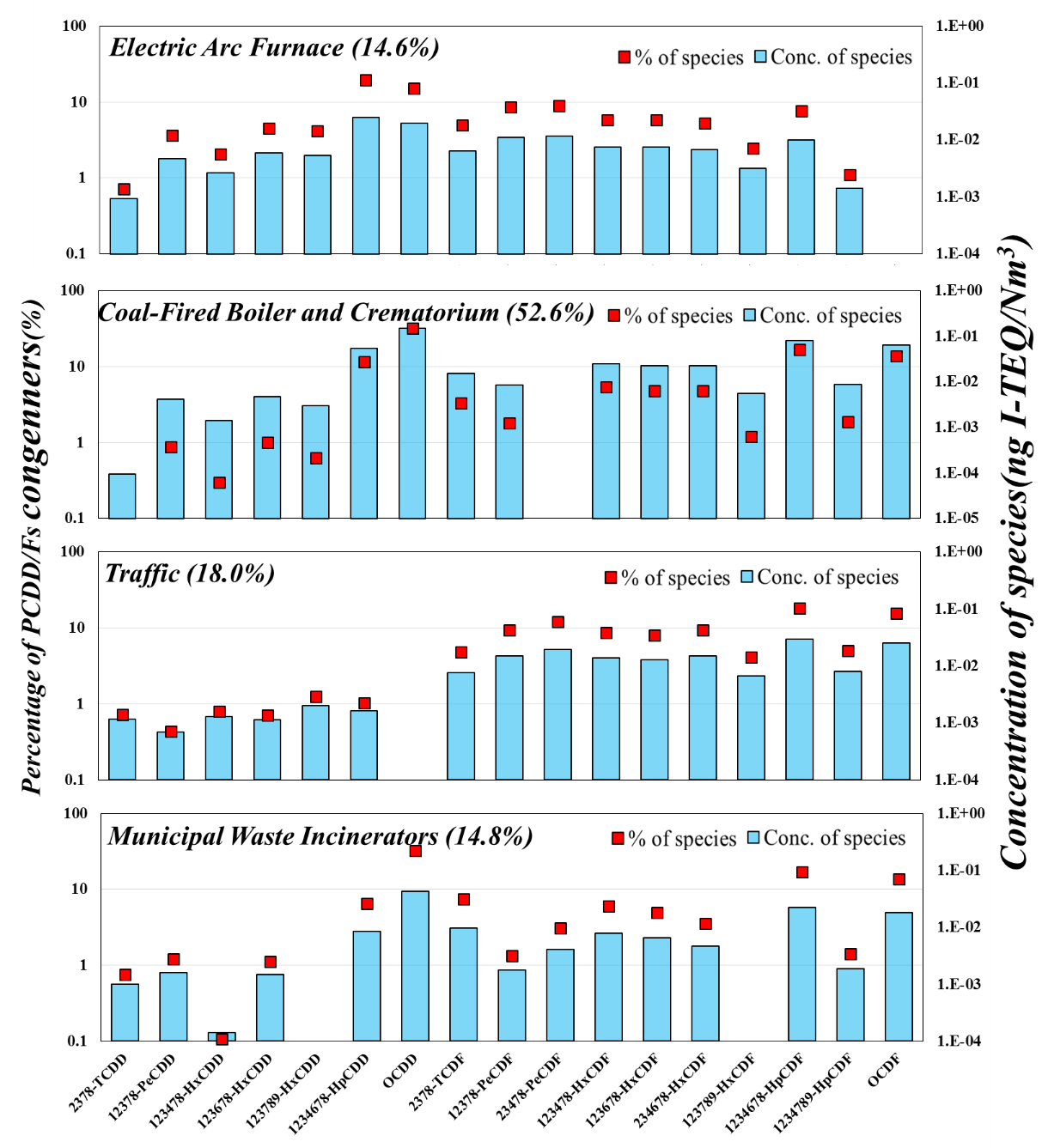


Fig.S6 Source apportionment of atmospheric PCDD/Fs in the vicinity of stationary pollution in Taiwan by using PMF.


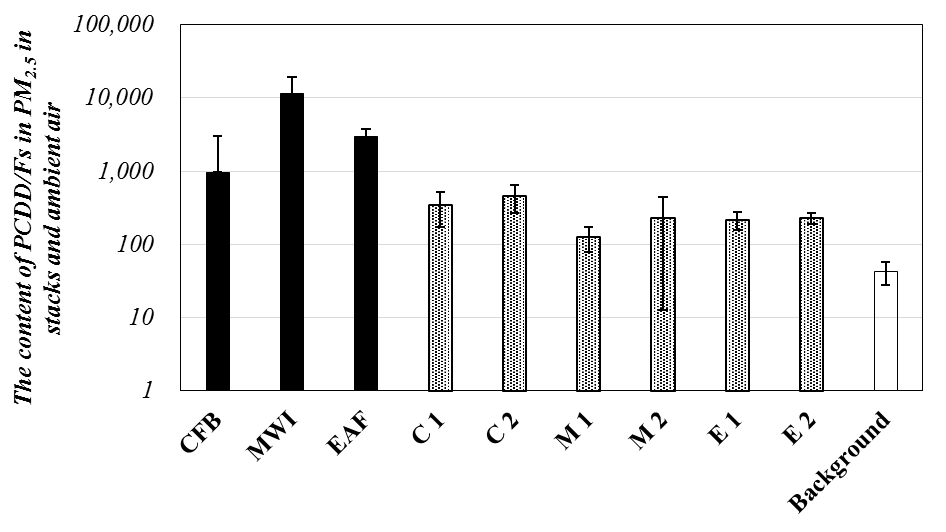


Fig.S7 The content of PCDD/Fs in PM_2.5_ in stacks and ambient air.
